# Defining the Innate Immune Responses for SARS-CoV-2-Human Macrophage Interactions

**DOI:** 10.3389/fimmu.2021.741502

**Published:** 2021-10-04

**Authors:** Mai M. Abdelmoaty, Pravin Yeapuri, Jatin Machhi, Katherine E. Olson, Farah Shahjin, Vikas Kumar, You Zhou, Jingjing Liang, Kabita Pandey, Arpan Acharya, Siddappa N. Byrareddy, R. Lee Mosley, Howard E. Gendelman

**Affiliations:** ^1^ Department of Pharmaceutical Sciences, College of Pharmacy, University of Nebraska Medical Center, Omaha, NE, United States; ^2^ Therapeutic Chemistry Department, Pharmaceutical and Drug Industries Research Division, National Research Centre, Giza, Egypt; ^3^ Department of Pharmacology and Experimental Neuroscience, College of Medicine, University of Nebraska Medical Center, Omaha, NE, United States; ^4^ Mass Spectrometry and Proteomics Core, University of Nebraska Medical Center, Omaha, NE, United States; ^5^ Center for Biotechnology, University of Nebraska-Lincoln, Lincoln, NE, United States; ^6^ Department of Population and Quantitative Health Sciences, School of Medicine, Case Western Reserve University, Cleveland, OH, United States

**Keywords:** macrophages, SARS-CoV-2, cytokine storm, interferon, end-organ disease, inflammation, transcriptomics, proteomics

## Abstract

Host innate immune response follows severe acute respiratory syndrome coronavirus 2 (SARS-CoV-2) infection, and it is the driver of the acute respiratory distress syndrome (ARDS) amongst other inflammatory end-organ morbidities. Such life-threatening coronavirus disease 2019 (COVID-19) is heralded by virus-induced activation of mononuclear phagocytes (MPs; monocytes, macrophages, and dendritic cells). MPs play substantial roles in aberrant immune secretory activities affecting profound systemic inflammation and end-organ malfunctions. All follow the presence of persistent viral components and virions without evidence of viral replication. To elucidate SARS-CoV-2-MP interactions we investigated transcriptomic and proteomic profiles of human monocyte-derived macrophages. While expression of the SARS-CoV-2 receptor, the angiotensin-converting enzyme 2, paralleled monocyte-macrophage differentiation, it failed to affect productive viral infection. In contrast, simple macrophage viral exposure led to robust pro-inflammatory cytokine and chemokine expression but attenuated type I interferon (IFN) activity. Both paralleled dysregulation of innate immune signaling pathways, specifically those linked to IFN. We conclude that the SARS-CoV-2-infected host mounts a robust innate immune response characterized by a pro-inflammatory storm heralding end-organ tissue damage.

## Introduction

Severe acute respiratory syndrome coronavirus 2 (SARS-CoV-2), the causative agent of coronavirus disease 2019 (COVID-19), is an enveloped positive-stranded RNA virus belonging to the *Coronaviridae* family, *Betacoronaviruses* genus (1). COVID-19 has posed an unprecedented global threat to public health, and in March of 2020, it was declared a pandemic by the World Health Organization (WHO) (2). COVID-19 ranges from asymptomatic infection to mild pneumonia and, in its most severe form, progression to acute respiratory distress syndrome (ARDS). Such pulmonary compromise is associated with dyspnea and hypoxia that can progress to severely compromised lung dysfunction and multiorgan system failure and death (3). Disease mortality is linked to cytokine storm syndrome (CSS), heralded by innate immune activation with the secretion of excessive pro-inflammatory cytokines (4). Indeed, nearly 15% of reported COVID-19 disease cases progress to ARDS defined by widespread inflammatory-associated lung tissue damage and multiorgan failure (5) involving heart, liver, gastrointestinal tract, kidney, and brain (6). Viral persistence in the face of such end-organ disease is linked to cell expression of angiotensin-converting enzyme 2 (ACE2), the molecule that SARS-CoV-2 utilizes for receptor-mediated cell entry (7).

Mononuclear phagocytes (MPs; monocytes, macrophages, and dendritic cells) are the governors of innate immunity serving to contain microbial infection (8). Immediately following viral exposure, the process of intracellular microbial removal is initiated through recognition of viral pathogen-associated molecular patterns (PAMPs) by pattern recognition receptors (PRRs). This includes, but is not limited to, cytosolic retinoic acid-inducible gene I (RIG-I)-like receptors (RLRs) and extracellular and endosomal toll-like receptors (TLRs). During the initiation of virus-cell interactions, secretion of pro-inflammatory cytokines and chemokines initiate intracellular killing, antigen presentation, and mobilization of adaptive immunity. Factors that are engaged include, but are not limited to, interleukin-6 (IL-6), IL-1, tumor necrosis factor-alpha (TNF-α), and C-X-C motif chemokine ligand 10 (CXCL10) (9). Type I interferon (IFN) α and β responses occur in tandem to control both viral replication and dissemination (10). During infection with SARS-CoV-2, CSS contributes to COVID-19-associated multiorgan failure (11, 12). In particular, infiltrating inflammatory MPs in lungs, heart, kidney, spleen, and lymph nodes are seen in post-mortem tissues of COVID-19-infected patients (13–16). Despite the critical role of MPs to clear the infection, these same cells underlie the pathobiology of COVID-19.

Mounting evidence confirms abortive MP infection by SARS-CoV-2 (17, 18). Nonetheless, such viral cell engagements are sufficient to induce activation and pro-inflammatory cytokine secretory responses. The nature of virus-MP interactions and their role in abortive viral infection and associated tissue pathologies remains enigmatic. To such ends, we pursued transcriptomic and proteomic analyses of immune cells following virus infection to identify modulations of host immune responses. We employed MPs to assess immune responses following SARS-CoV-2 cell engagements for cell activation using human immune response arrays and mass spectrometry-based label-free proteomic quantification methods. These techniques serve to define virus-induced innate immune responses linked to antiviral immunity. Based on these tests, we have uncovered the dysregulation of a spectrum of viral-induced responses related to IFN signaling pathways, complement activation, and linked adaptive immune responses. All provide unique insight into how the inflammatory response occurs as a consequence of SARS-CoV-2 infection. Virion persistence for up to 14 days after viral exposure despite restrictive infection underlies the persistence of immune activation. Most importantly, the data provides a signature for virus-induced disease pathobiology, including COVID-19-associated CSS and multiorgan dysfunction. All affect the most severe disease morbidities and mortalities seen as a consequence of viral exposure, transmission, and dissemination.

## Materials and Methods

### Isolation and Cultivation of Human Monocytes

Human monocytes were obtained by leukapheresis from hepatitis B and HIV-1/2 seronegative donors and purified by counter-current centrifugal elutriation (19). Monocytes were seeded in 6-well plates (3x10^6^ cells/well) in Dulbecco’s Modified Eagle’s Media (DMEM) containing 4.5 g/l glucose, L-glutamine, and sodium pyruvate, and supplemented with 10% heat-inactivated human serum, 50 μg/ml gentamicin, 10 μg/ml ciprofloxacin, and 1000 U/ml human macrophage colony-stimulating factor (M-CSF) to facilitate differentiation of monocytes into monocyte-derived macrophages (MDMs). Cells were incubated at 37°C with 5% CO_2,_ and the culture medium was half-exchanged with fresh medium every other day (20).

### Flow Cytometry Assays

Human monocytes were evaluated by flow cytometry for levels of SARS-CoV-2 cell entry receptor ACE2 and monocyte-macrophage phenotypic surface markers, CD14 and CD16, during macrophage differentiation with or without captopril (Sigma-Aldrich, C4042). Captopril was added to the culture medium to increase ACE2 cell expression. On days 0, 1, 3, 5, and 7 during differentiation, monocytes-macrophages were stained with fluorescently-conjugated antibodies to detect human ACE2 (APC, LSBio, LS−C275129, polyclonal), CD14 (Alexa Fluor 488, eBioscience, clone 61D3), and CD16 (PE, eBioscience, eBioCB16, clone CB16), and with isotype-matched antibodies serving as negative controls. Stained cells were examined with an BD LSR II flow cytometer (BD Biosciences) and analyzed using BD FACSDiva software.

### SARS-CoV-2 Infection

Experiments involving SARS-CoV-2 were performed in the University of Nebraska Medical Center (UNMC) biosafety level 3 (BSL-3) core facility and approved by UNMC Institutional Biosafety Committee (IBC) (protocol number 20-05-027-BL3). The SARS-CoV-2 strain used in this study was isolate USA-WI1/2020 (BEI, NR-52384) unless stated otherwise. The virus was passaged on Vero.STAT1 knockout (KO) cells (ATCC, CCL-81-VHG) and titer was determined by plaque assay in Vero E6 cells (ATCC, CRL-1586) (21). The selection of multiplicity of infection (MOI) for the following experiments was made based on a pilot experiment using isolate USA-WA1/2020 (BEI, NR-52281). Human MDMs were challenged at increasing MOIs of 0.001, 0.01, and 0.1. The SARS-CoV-2 and mock-treated MDMs and culture fluids were collected from days 1 to 11 after viral exposures. Total RNA was isolated using RNeasy Mini Kit (Qiagen, 74104), and cDNA was generated utilizing RevertAid First Strand cDNA synthesis kit (Thermo Fisher Scientific, K1622) followed by amplification and quantification using RT^2^ Profiler Human Innate and Adaptive Immune Response 96-well Array (Qiagen, 330231) with RT^2^ SYBR Green ROX qPCR Mastermix (Qiagen, 330523). The qPCR cycling conditions were 95°C for 10 minutes for 1 cycle, followed by 40 cycles of 95°C for 15 seconds and 60°C for 1 minute using Eppendorf Mastercycler ep realplex 2S. Fold changes were determined by Qiagen’s RT^2^ profiler analysis software (version 3.5). Based on the transcriptomic data of day 11 after viral exposure, the viral exposure was repeated using MDMs isolated from different donors with the WA1/2020 viral strain administered at an MOI of 0.01. The transcriptomic profile of SARS-CoV-2 and mock-treated MDMs was assessed on day 4 after viral exposure. Based on the transcriptomic data of day 4, the MOI of 0.01 was selected. Five days following cell cultivation MDMs were exposed to the WI1/2020 viral strain at an MOI of 0.01. Exposure was maintained with or without captopril, and the virus-cell mixtures incubated at 37°C in 5% CO_2_ with shaking at 15-minute intervals for 1 hour. This was followed by incubation with the virus for an additional 3 hours. At the termination of viral cell incubation, the virus inoculum was removed, and cells were washed 3 times with phosphate-buffered saline (PBS). Mock-challenged cells were treated with a culture medium alone. Culture supernatants were collected at defined time points then used for viral quantitative reverse transcription-polymerase chain reaction (RT-qPCR) and type I IFN activity assays. Vero.STAT1 KO cells were maintained for study based on their high susceptibility to virus infection due to lack of Signal Transducer and Activator of Transcription 1 (STAT1) protein required for cellular antiviral responses (22). Thus, the SARS-CoV-2 replication kinetics in MDMs were compared against viral infection in Vero.STAT1 KO cells that were monitored over 5 days post-viral exposure.

### RT-qPCR Assay

Total RNA was extracted from the infected MDMs on days 1, 3, 5, 7, and 10 following viral exposure, followed by quantification for SARS-CoV-2 genome equivalents by RT-qPCR, and the gene expression was normalized to HPRT1 (Thermo Fisher Scientific, 4331348). Forward primer: 5’ GGACTAATTATGGACAGGACTGAA 3’, Reverse primer: 5’ ACAGAGGGCTACAA TGTGATG 3’, and Probe: 5’ FAM-AGATGTGATGAAGGAGATGGGAGGC-BHQ-1 3’and presented as fold change in gene expression of infected cells relative to mock-challenged cells. After viral exposure, culture supernatants were collected on days 1, 3, 5, 7, and 10. Viral RNA was extracted using QIAamp Viral RNA Mini Kit (Qiagen, 52906). SARS-CoV-2 genome equivalents were quantified in culture supernatant by RT-qPCR using 2019-nCoV CDC probe and Primer Kit for SARS-CoV-2 (Biosearch Technologies, KIT-nCoV-PP1-1000). Forward primer: 5’ GACCCCAAAATCAGCGAAAT 3’, Reverse primer: 5’ TCTGGTTACTGCCAGTTGAATCTG 3’, and Probe: 5’ FAM-ACCCCGCATTACGTTTGGTGGACC-BHQ-1 3’. RT-qPCR was performed using Taqman Fast Virus 1-step Master Mix (Thermo Fisher Scientific, 4444434) in StepOne Plus real-time PCR thermocycler (Applied Biosystems) using the following cycling conditions: 50°C for 10 minutes, 95°C for 3 minutes, and 40 cycles of 95°C for 15 seconds, followed by 60°C for 1 minute. The SARS-CoV-2 genome equivalent copies were calculated using control RNA from heat-inactivated SARS-CoV-2 USA-WA1/2020 (BEI, NR-52347).

### Transmission Electron Microscopy

For negative staining analysis of viral particles used for cell infection experiment, purified SARS-CoV-2 viruses were de-activated and fixed in 2% glutaraldehyde and 2% paraformaldehyde (PFA) in 0.1 M Sorenson’s phosphate buffer for 1 hour at room temperature. Briefly, for negative staining, a drop of the virus sample in a fixative solution was placed onto a formvar and carbon-coated grid for 30-40 seconds. After removing excess sample solution with filter paper, the grid was placed with sample side down on a drop of 1% phosphotungstic acid (PTA) in water, stained for 30 seconds, then excess PTA solution was blotted with filter paper. Stained samples were examined and imaged using a Hitachi H7500 TEM (Hitachi High-Tech GLOBAL) and a bottom-mount AMT camera (AMT Imaging). For ultrastructural analysis, mock and SARS-CoV-2-challenged MDMs sampled at 1, 3, 5 and 14 days post-viral inoculation were washed 2 times with PBS and fixed in a solution of 2% glutaraldehyde and 2% PFA in 0.1 M Sorenson’s phosphate buffer for 24 hours at 4°C which have been washed 3 times with PBS to clear excess fixative solution. TEM analysis was performed as previously described (23) in samples post-fixed in a 1% aqueous solution of osmium tetroxide for 30 minutes that were dehydrated in 50, 70, 90, 95, and 100% graded ethanol. Spurr’s resin was used as embedding medium after solvent transition with ethanol and Spurr’s resin (50:50 ethanol:resin, followed by twice immersion in 100% Spurr’s resin for 2-3 hours for each solution), and embedded samples were cured at 60 - 65°C for 24 hours. Ultrathin sections (100 nm) were cut with Leica UC7 ultramicrotome, placed on 200-mesh copper grids, followed by staining with 2% uranyl acetate and Reynold’s lead citrate, and examined with a Hitachi H7500 TEM at 80 kV. Images were acquired digitally with an AMT digital imaging system.

### Transcriptomic Analyses

SARS-CoV-2 and mock-challenged MDMs were collected on days 1, 3, and 5. Total RNA was isolated, and cDNA was synthesized, then amplification and quantification using an RT^2^ Profiler Human Innate and Adaptive Immune Response 96-well Array, and fold changes were determined by Qiagen’s RT^2^ Profiler analysis software. Ingenuity Pathway Analysis (IPA) (Qiagen) was used to identify the pathways and networks affected post-viral exposure. Functional and pathway enrichment analyses of screened genes in SARS-CoV-2-challenged MDMs were compared to mock-challenged MDMs at different time intervals following viral exposure. Gene ontology (GO) annotation was conducted with GO Resource database (http://geneontology.org). The Kyoto Encyclopedia of Genes and Genomes (KEGG) pathway enrichment analysis was conducted utilizing DAVID (http://david.abcc.ncfcrf.gov/) (24), an online tool providing a comprehensive set of functional annotations providing biological meaning to the gene lists. The gene set enrichment analysis (GSEA) was conducted using ReactomeFIViz (https://reactome.org/tools/reactome-fiviz) (25), a Cytoscape application for pathway and network-based data analyses. The Search Tool for the Retrieval of Interacting Genes/Proteins (STRING) local network cluster enrichment was conducted using the STRING database (http://string-db.org), which provides critical assessments and integration of protein-protein interaction (PPI). This includes direct (physical) and indirect (functional) associations in a given organism (26).

### Measures of IFN Activity

MDBK cells (ATCC, CCL-22) were cultured in Eagle’s Minimum Essential Medium (EMEM) containing 10% heat-inactivated fetal bovine serum (FBS) and 50 µg/ml gentamicin (27). Vesicular stomatitis virus (VSV Indiana laboratory strain) (V-520-001-522, ATCC, VR-1238) was passaged on Vero cells (ATCC, CCL-81), and viral titer was determined using the plaque assay in Vero cells. MDBK cells bind and respond to human type I IFNs, α and β, but not IFN-gamma (IFN-γ) (27). Culture supernatants were collected from virus-exposed MDMs on days 1, 3, and 5 following infection with SARS-CoV-2 at MOI of 0.01, then assessed for IFN-α/β activity by protection against the VSV-induced cytopathicity measured in MDBK cells (27). Polyinosinic-polycytidylic acid (poly(I:C); Sigma-Aldrich, P9582) served as a positive control for IFN induction *via* TLR3 engagement (28). Recombinant human IFN-α (PBL Assay Science, 11200-2) was used as assay standard. We also investigated IFN activity upon the challenge of Teflon flask-suspended monocytes with SARS-CoV-2 (MOI=0.01), before and after treatment of cells with 100 µg/ml poly(I:C).

### Western Blot Analysis

At days 1, 3, and 5 after infection, MDMs were collected, and total protein was extracted using lysis buffer containing 0.1% SDS, 100 mM Tris-HCL, 150 mM NaCl, 1 mM EDTA, 1% Triton X-100, supplemented with protease and phosphatase inhibitor cocktail (Sigma-Aldrich, PPC1010). Protein concentration was determined utilizing Pierce 660 Protein Assay kit (Thermo Fisher Scientific, 22662) with ionic detergent compatibility reagent (Thermo Fisher Scientific, 22663) following the manufacturer’s instructions. Protein lysates (25 μg) were resolved by SDS-PAGE and transferred to Immobilon-P PVDF membrane (Sigma-Aldrich, IPVH00010). Membranes were blocked in 5% nonfat milk in TBST buffer at room temperature for 1 hour, followed by incubation with primary antibodies to IFN-α (1:500, Thermo Fisher Scientific, MA5-37518), IFN-β (1:1000, Abcam, ab85803), and β-actin (1:3000, Sigma-Aldrich, A3854) at 4°C overnight, followed by 1 hour incubation in 3% nonfat milk in Tris-buffered saline with 0.1% Tween 20 detergent (TBST) buffer with horseradish peroxidase-conjugated anti-rabbit (1:2000, R&D Systems, HAF008) or mouse (1:2000, R&D Systems, HAF018) secondary antibody. Immunoreactive bands were detected using SuperSignal West Pico Chemiluminescent substrate (Thermo Fisher Scientific, 34080), and images were captured using an iBright CL750 Imager (Thermo Fisher Scientific). Immunoblots were quantified using ImageJ software (NIH) relative to β-actin expression.

### Proteomic Analysis

On days 1, 3, and 5 post-infection, MDMs were collected, lysed with 2% SDS in 100 mM Tris-HCL and 100 mM dithiothreitol, pH 7.6, and supplemented with protease and phosphatase inhibitors. Protein concentration was determined using Pierce 660 Protein Assay kit with ionic detergent compatibility reagent according to the manufacturer’s instructions. Afterward, samples were processed as previously described (29) using filter-aided sample preparation (FASP, Pall Life Sciences, OD010C34) to digest 50 μg per sample. Following overnight digestion, samples were cleaned using the Oasis MCX column (Waters, 186000252) and C18 Zip-Tips (Sigma-Aldrich, ZTC18M960). Cleaned peptides were quantitated using NanoDrop2000 at 205 nm. Following resuspension in 0.1% formic acid, 2 μg of the sample was used for label-free quantification (LFQ) in UNMC Mass Spectrometry and Proteomics Core Facility as previously described (30). Protein identification was performed by searching MS/MS data against the swiss-prot homo sapiens and SARS-CoV-2 protein databases downloaded on April 2021 and August 2021, respectively, using the in-house PEAKS X + DB search engine. The search was set up for full tryptic peptides with a maximum of 2 missed cleavage sites. Acetylation of protein N-terminus and oxidized methionine were included as variable modifications, and carbamidomethylation of cysteine was set as fixed modification. The precursor mass tolerance threshold was set 10 ppm for, and the maximum fragment mass error was 0.02 Da. The significance threshold of the ion score was calculated based on a false discovery rate (FDR) of ≤ 1%. Quantitative data analysis was performed to determine differentially expressed proteins between mock and SARS-CoV-2-challenged MDMs at different time points after viral exposures using Progenesis QI Proteomics 4.2 (Nonlinear Dynamics). Statistical analysis was performed using ANOVA and FDR for pathway analysis. This was controlled using the Benjamini-Hochberg (BH) method (31). A protein was differentially expressed if p value ≤ 0.05 and the absolute value of fold change ≥ 2. Functional and pathway enrichment analysis of differentially expressed proteins in SARS-CoV-2-challenged MDMs compared to mock-challenged MDMs was conducted at different time points after the virus challenge. Gene enrichment analysis to identify immune system processes affected after virus exposure was performed using Cytoscape in conjunction with the plug-in ClueGO (32). GO annotation, KEGG, Reactome GSEA, and STRING analyses were conducted using GO Resource, DAVID, ReactomeFIViz, and STRING databases, respectively. Overlapping genes between transcriptomic and proteomic data were identified and represented in Venn diagrams.

### Correlation Analysis of Transcriptomic and Proteomic IFN Pathway Genes

The parametric Pearson’s product moment correlation analysis between transcriptomic and proteomic data for day 1, 3, and 5-time points were conducted for 10 IFN pathway-related genes. The correlation coefficient (r) for each gene was calculated.

### Statistical Analysis

Results are presented as the mean ± standard error of the mean (SEM). Student’s t-test or one-way ANOVA followed by Tukey’s multiple comparison test was used to analyze differences in the mean values between groups. P values ≤ 0.05 were considered statistically significant. Statistical analysis was performed using GraphPad Prism 9.1.0 software (GraphPad Software, San Diego, CA).

## Results

### ACE2 Receptor Expression During Human Monocyte-Macrophage Maturation

**Figure 1:**
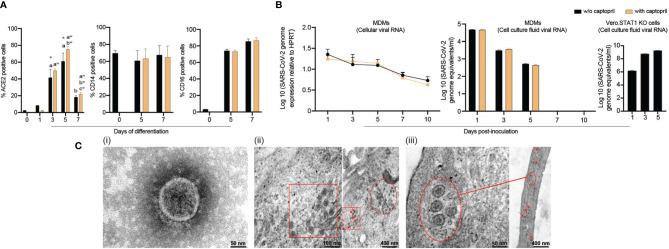
SARS-CoV-2 restrictive infection of human MDMs. **(A)** Expression of SARS-CoV-2 cell entry receptor ACE2 and phenotypic surface markers CD14 and CD16, during differentiation of monocytes into macrophages, was analyzed by flow cytometry in absence or presence of captopril. **(B)** SARS-CoV-2 presence in MDMs. SARS-CoV-2 (MOI=0.01) was used to infect MDMs (with or without captopril). The number of virus genome equivalents per ml was measured in cell lysates and culture supernatants by RT-qPCR. Vero.STAT1 KO cells served as a positive control. **(C)** Transmission electron micrographs of SARS-CoV-2-challenged MDMs. (**C**.i) A viral particle from a pool of SARS-CoV-2 used for the virus challenges. SARS-CoV-2-challenged MDMs [(**C.**ii) day 5, and (**C.**iii) day 14 after viral exposure]. Red arrows, circles, and boxes demonstrate clusters of viral particles within the virus-challenged macrophages. All experiments were done at least twice with representative images depicted here. **(A, B)** Data are represented as mean ± SEM (n=3-6 donors). Statistical significance between groups was determined using one-way ANOVA, and p < 0.05 was considered significant (*significantly different from day 0 w/o captopril, a: significantly different from day 1 w/o captopril, b: significantly different from day 5 w/o captopril, a^w^: significantly different from day 1 with captopril, b^w^: significantly different from day 3 with captopril, c^w^: significantly different from day 5 with captopril). w/o: without. Scale bars: 50 nm (i and left panel of iii), 100 nm (left panel of ii), and 400 nm (right panels of ii and iii).

ACE inhibitors were used to upregulate the SARS-CoV-2 receptor ACE2 and potentiate cell entry (33–35). As ACE inhibitors are frequently prescribed to treat hypertension, we posit that they can increase susceptibility to SARS-CoV-2. Therefore, we measured ACE2 expression on freshly isolated monocytes throughout cell isolation and differentiation. Days 1, 3, 5, and 7 were evaluated for cells cultured with or without captopril, an ACE inhibitor which is thought to increases ACE2 expression. In these studies, ACE2 expression by monocytes was found to peak by day 5 after initiation of cell differentiation then decreased after that (**Figure 1A**), which corresponds to the susceptibility of infection of monocytes-macrophages by other viruses (20, 36, 37). On day 5, ACE2 expression increased by 23% in captopril-treated cells (p = 0.24). In parallel, the expression of phenotypic monocyte-macrophage surface markers, CD14 and CD16, were not changed (**Figure 1A**). Representative flow cytometry histograms for ACE2, CD14, and CD16 expression are shown in **Supplementary Figure 1**. Based on these observations, we challenged MDMs with SARS-CoV-2 on day 5 after the start of cell differentiation and assessed whether captopril affects susceptibility to infection.

### SARS-CoV-2 MOI Determinations

To select the MOI of 0.01, pilot experiments were done screening several infectious parameters. MOIs of 0.001, 0.01, and 0.1 were chosen in these experiments to challenge the MDMs with consequent monitoring of signs of productive infection. The “putative” growth of the SARS-CoV-2 in MDMs was then determined by evaluating the culture supernatants over 11 days after viral exposures. At varied MOIs, the number of genome copies remained unchanged over time. This indicated restrictive viral infection without evidence of viral replication (**Supplementary Figure 2**). However, the transcriptomic profile of SARS-CoV-2-challenged MDMs (MOI=0.01) showed more than 5-fold increase in gene expression of pro-inflammatory cytokines and chemokines up to day 11. This included *IL1B*, *IL1R1*, chemokine (C-C motif) ligand 8 (*CXCL8*), and *CCL2* (78.56, 40.11, 6.48, 81.33-fold, respectively) (**Supplementary File 1**). The MOI used for these experiments were held constant at 0.01. For these analyses, we challenged MDMs with SARS-CoV-2 then analyzed the cell’s transcriptomic profile on day 4 after viral exposure. In this replicate experiments, a more than 2-fold increase in expression of *IL17A*, *IL18*, *IL1B*, *CXCL8*, *NLRP3*, and *TLR8* was recorded (p = 0.39, 0.27, 0.34, 0.28, 0.18, 0.34, respectively) (**Supplementary File 2**). Additionally, the data showed, in parallel, a more than 2-fold increase in select IFN-related genes that included *IFNA1*, *IFNGR1*, *IRF3*, IFN regulatory factor 7 (*IRF7*), and tyrosine kinase 2 (*TYK2*) (p = 0.1, 0.19, 0.35, 0.26, and 0.29, respectively) (**Supplementary File 2**). Based on these observations, we confirmed the MOI of 0.01 in eliciting a cell-based activation profile.

### Persistence of Viral Components and Virions in MDMs Is Established Following SARS-CoV-2 Exposure

To investigate whether human MDMs are susceptible to SARS-CoV-2 infection, monocytes were isolated from healthy donors and differentiated in the presence or absence of captopril and infected at day 5 after cell culture. The kinetic growth of SARS-CoV-2 in MDMs was determined from in cell lysates and culture supernatant tests of challenged cells over 10 days post-infection. In the cell lysates (captopril-treated and controls), the SARS-CoV-2 genome equivalent copies were not altered up to 10 days after MDM viral exposures (**Figure 1B**). In all studied groups (captopril-treated and controls), the SARS-CoV-2 genome equivalent copies in the supernatant significantly decreased during 5 days of viral exposure, becoming undetectable after that (**Figure 1B**). Additionally, the number of genome copies did not change significantly with or without captopril. These results demonstrated that SARS-CoV-2 infection of human MDMs was restrictive without evidence of viral replication and differentiated monocytes could not generate progeny virus in contrast to productive infection of SARS-CoV-2 in Vero.STAT1 KO cells (**Figure 1B**). Our data also indicated that captopril did not alter the susceptibility of MDMs to SARS-CoV-2 infection.

### Ultrastructural Features of SARS-CoV-2-Challenged MDMs

Viral particles and mock and SARS-CoV-2-challenged MDMs were fixed at different time points after viral exposure and then examined by TEM to determine ultrastructural changes in the virus-challenged cells (**Figure 1C.**i-iii****). Negative stain TEM shows the ultrastructure of a viral particle from a pool of SARS-CoV-2 used for the virus challenges (**Figure 1C.**i****). By day 5 after exposure, a cluster of viral particles was shown in the challenged macrophages (**Figures 1C.**ii****). Surprisingly, while no viral genome copies were detected after 5 days (**Figure 1B**), mature virions were observed 14 days after initial exposure (**Figure 1C.**iii****). These underlie the persistence of virus in macrophages despite evidence of restrictive infection.

### Persistent SARS-CoV-2 Components in MDMs Induce Pro-Inflammatory Factors, but Not IFN

**Figure 2:**
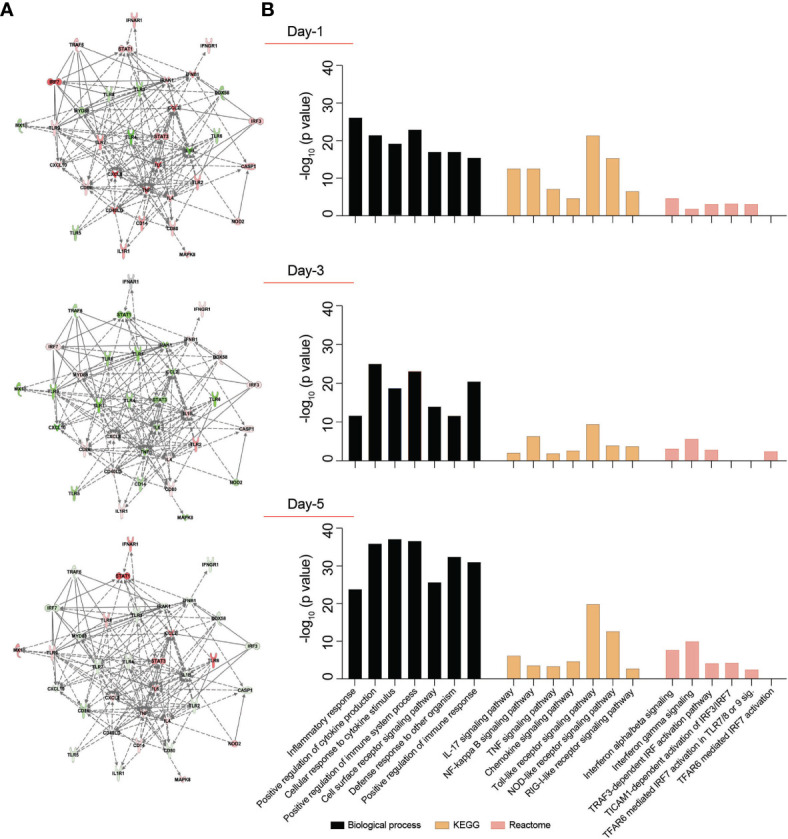
Transcriptomic profile of SARS-CoV-2-challenged MDMs. Expression of 84 genes specific for human innate and adaptive immune responses were screened in SARS-CoV-2-challenged MDMs compared to mock-challenged MDMs at different times post-challenge, using RT^2^ Profiler Human Innate and Adaptive Immune Response 96-well Array. Fold changes in the gene expression were determined *via* Qiagen’s RT^2^ Profiler analysis software (n=4 donors). **(A)** IPA was performed with upregulated or downregulated genes to identify putative network interactions involved in SARS-CoV2-MDM interactions. Upregulated genes are shaded red with darker shade indicating higher upregulation, while green shades denote downregulation in gene expression. Solid grey lines indicate direct interactions, while dotted grey lines correspond to indirect interactions. **(B)** Functional and pathway enrichment analysis of transcriptomic dataset was performed using GO-term, KEGG, and Reactome analyses at different time points post-infection. Different immune and inflammation-related biological processes and pathways affected in SARS-CoV-2-challenged MDMs were plotted as a bar chart compared to mock-challenged MDMs.

To investigate alterations in macrophage-mediated innate immune responses after SARS-CoV-2 exposure, a total of 84 key genes were examined. Transcriptional changes linked to immunity were screened using RT^2^ Profiler Human Innate and Adaptive Immune Response Array. Expression of immune response genes in SARS-CoV-2-challenged MDMs were investigated against control “mock-challenged cells” on days 1, 3, and 5 (**Supplementary Figure 3** and **Supplementary File 3**). On day 1, more than 2-fold increase in expression of pro-inflammatory cytokine and chemokine genes were detected, including *IL-6* (p = 0.32)*, TNF-α* (p = 0.32)*, IL-1α* (p = 0.98)*, IL17A* (p = 0.38)*, IL8* (p = 0.08), colony-stimulating factor 2 (*CSF2*) (p = 0.37), chemokine (C-C motif) ligand 2 (*CCL2*) (p = 0.32), and *CCL5* (p = 0.32). Interestingly, on day 3, gene expression of the node-like receptor family pyrin domain containing 3 (*NLRP3*) and *IL-1β* increased by 8.84- and 5.64-fold, respectively (p = 0.98 and 0.78, respectively). On day 5, expression for these pro-inflammatory cytokines and chemokines were at baseline or decreased with the exception of *IL-6* and *CCL2* (p = 0.91 and 0.65, respectively). Notably, on day 5 after viral exposure there was an upregulation of *STAT1* gene expression (4.1-fold, p = 0.71), *IFNAR1* gene expression (2.64-fold, p = 0.17), and *MX1* as an IFN-stimulated gene (2.68-fold, p = 0.56). MX1 has been shown to have wide antiviral activity against RNA and DNA viruses by its direct effect on the viral ribonucleoprotein complex and its GTPase activity essential for SARS-CoV-2 replication (38). Additionally, on day 5 after viral exposure, there was a slight upregulation of *TLR8* gene expression (1.68-fold, p = 0.41), supporting the pro-inflammatory environment in SARS-CoV-2-challenged MDMs (39) sustained to day 5 after viral exposures. There are dual regulatory activities between inflammatory and a compensatory anti-inflammatory response as shown through *IL-10*, *IL-4*, and *IL-13.* This dual regulatory phenotype of the macrophages is depicted with shown data sets for *IL-4* (7.82-fold, p = 0.095) and *IL-13* (7.06-fold, p = 0.066) (40, 41). Such gene expression for this class of cytokines was shown at day 4 following the viral exposure (**Supplementary File 2**). Moreover, the gene expression of anti-inflammatory cytokine *IL-10* was elevated on days 1 and 5 (3.1 and 3.62-fold, p = 0.34 and 0.22, respectively), supporting the attenuation of pro-inflammatory responses. Importantly, expression of IFN-linked genes *IFN-α1, IFN-β1, IFN-γ*, IRF7, and TYK2 genes increased more than 2-fold (p = 0.18, 0.38, 0.35, 0.69, and 0.38, respectively) on day 1 but returned to near baseline by days 3 and 5. We posit that non-significant p values underlie the divergent clinical responses to SARS-CoV-2 infection. As can be observed we used the identical experimental approach with different donors and viral strains and while each showed an upregulation of pro-inflammatory cytokine and chemokine gene expression the levels recorded were variable dependent on each of the variables. Those variables included the donor, the virus and the time after exposures. These data demonstrate a complex pro- and anti-inflammatory network interactions that are sustained to day 5 with attenuated IFN responses following SARS-CoV-2 exposure (**Figure 2A**).

### Functional and Pathway Enrichment of Immune Regulated Genes

SARS-CoV-2 infection may induce dynamic changes of immune-based gene expression in specific cellular biological processes and pathways in virus-challenged cells. To assess these changes, functional and pathway enrichment analyses were performed on immune-regulated genes screened in SARS-CoV-2-challenged MDMs compared to controls at days 1, 3, and 5 (**Supplementary File 4**). Multiple immune processes were enriched upon viral exposure, including defense response to other organism, positive regulation of immune system processes, cell surface receptor signaling pathways, and positive regulation of immune responses (**Figure 2B**). All indicated they comprised activated immune responses in the virus-challenged MDMs. In addition, a series of inflammation-related processes were enriched, including inflammatory response, positive regulation of cytokine production, and cellular response to cytokine stimulus (**Figure 2B**), highlighting inflammation responses induced after viral exposure. Similarly, different immune and inflammation-related molecular functions such as cytokine activity, chemokine activity, complement component C1q binding, type I IFN receptor binding, and IL-1 receptor binding were enriched after virus exposure (**Supplementary File 4**). Furthermore, KEGG pathway analysis showed enrichment of TLR, NOD-like receptor, and RLR signaling pathways (**Figure 2B**), suggesting the activation of PRRs on MDMs to recognize viral PAMPs initiating innate immune responses. Moreover, KEGG pathway analysis depicted enrichment of IL-17, NF-κB, TNF, and chemokine signaling pathways (**Figure 2B**), highlighting different inflammatory pathways induced in the SARS-CoV-2-challenged MDMs. Interestingly, Reactome analysis revealed enrichment of multiple IFN-related signaling pathways, including IFN-α/β signaling, IFN-γ signaling, TICAM1-dependent activation of IRF3/IRF7, TRAF3-dependent IRF activation pathway, and TRAF6 mediated IRF7 activation (**Figure 2B**). The interacting proteins involved in the screened innate and adaptive immune-related genes were also identified using STRING analysis of their interaction networks (**Supplementary File 4**).

### SARS-CoV-2 Monocyte-Macrophage Engagement and Failed Induction of IFN Activity

**Figure 3:**
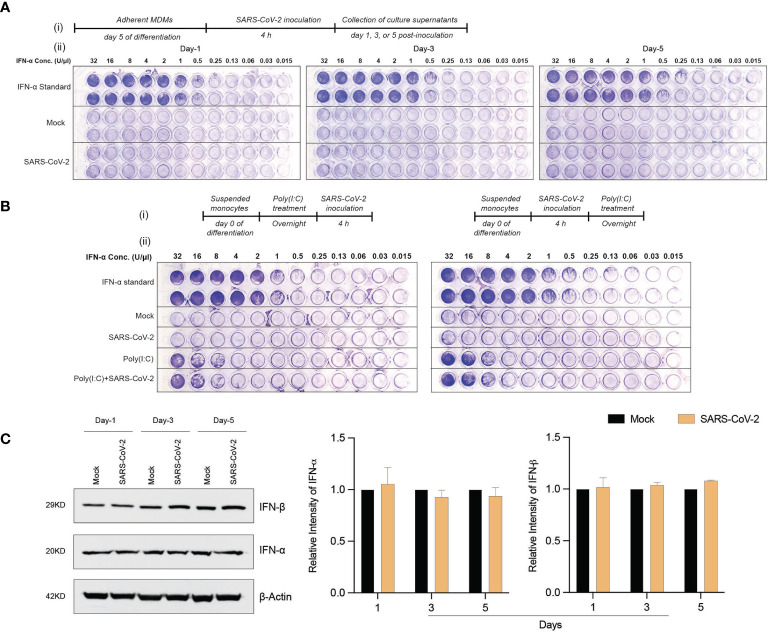
SARS-CoV-2 does not induce IFN activity in monocytes-macrophages. **(A)** MDMs were infected with SARS-CoV-2 (MOI=0.01) and the culture supernatants were collected at days 1, 3, and 5 post-infection. The collected culture supernatants were used to assess IFN activity in VSV-challenged bovine MDBK cells. Recombinant human IFN-α was used as standard (64 U/μl). 2-fold dilution was used for different conditions. (**A.**i) Experimental timeline. (**A.**ii) Representative images of IFN activity assay plates. **(B)** No additive or synergistic responses in poly(I:C)-induced IFN activity between control and SARS-CoV-2-challenged monocytes. Suspended monocytes were treated with 100 μg/ml poly(I:C) overnight before or after the infection with SARS-CoV-2 (MOI=0.01) for 4 h. (**B.**i) Experimental timeline. **(B**.ii**)** Representative images of IFN activity assay plates. **(C)** Western blot analysis was performed to determine expression of IFN-α and IFN-β in cell lysates at different times after viral exposure. Representative immunoblot and densitometric quantification are shown. All experiments were done at least twice, and one representative image is shown. **(C)** Data represent mean ± SEM (n=3 donors). Statistical significance between the groups was determined with unpaired Student’s t-test and p < 0.05 was considered significant.

To determine whether exposure to the SARS-CoV-2 can trigger antiviral IFN activities in human macrophages, culture supernatants were collected at different times post-viral exposure and assayed in VSV-challenged MDBK cells. Notably, no type I IFN activity was observed in SARS-CoV-2-challenged MDM cultures at different time intervals following viral challenge (**Figure 3A**). These data stand in contrast to the transcriptomic results, which displayed increases in IFN pathway-linked genes. Furthermore, no additive or synergistic IFN activity was detected in the control and SARS-CoV-2-challenged monocytes treated with poly(I:C) (**Figure 3B**). Taken together, the data demonstrate that SARS-CoV-2-MDM interactions do not affect IFN activities. To affirm this, we evaluated the protein expression level of IFN-α and IFN-β in SARS-CoV-2 exposed MDMs at days 1, 3, and 5 post-exposure. Similar to IFN activity, no IFN-α or IFN-β proteins were produced in the SARS-CoV-2-challenged MDMs more than mock-challenged cells (**Figure 3C**). Altogether, our data demonstrate that SARS-CoV-2 exposure of MDM does not trigger IFN activity or protein production in human monocytes-macrophages.

### Proteomic Profiles of SARS-CoV-2-Challenged MDMs

**Figure 4:**
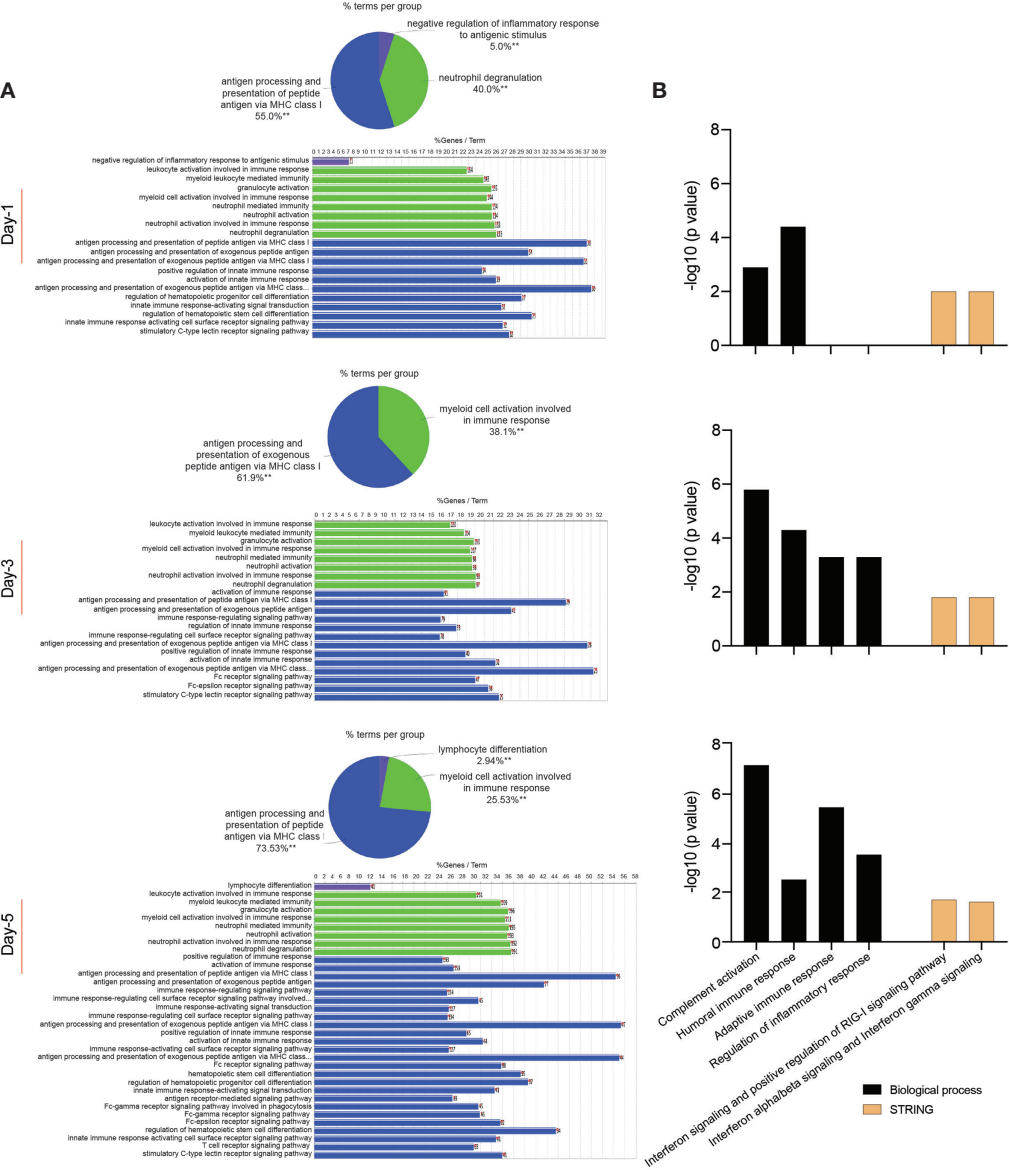
Proteomic profile of SARS-CoV-2-challenged MDMs. **(A)** Gene enrichment analysis was performed using Cytoscape in conjunction with the plug-in ClueGO at different times after viral exposure. Pie charts representing the distribution of identified differentially expressed proteins according to observed immune processes and bar charts demonstrating the specific processes that correspond to their classification. The same color key used in the pie charts is applied in the bar charts. **(B)** Functional and pathway enrichment analyses of proteomic dataset were performed using GO annotation, KEGG, Reactome GSEA, and STRING analyses at different time points post-infection. Different immune-related biological processes and PPI networks affected in SARS-CoV-2-challenged MDMs were plotted as a bar chart compared to mock-challenged MDMs.

In attempts to elucidate the mechanisms of the MDM response against the SARS-CoV-2 challenge, we obtained the proteome profiles from lysates of viral exposed macrophages and compared those to non-exposed counterparts using the homo sapiens proteome. On defined days after viral exposure, the expression of 4049, 4296, and 4246 proteins were identified and quantified using differential proteomic tests on days 1, 3, and 5, respectively (**Supplementary File 5**). Amongst total identified proteins, 1776, 1372, and 2448 proteins were significantly differentially expressed (p ≤ 0.05 and fold change ≥ 2), on days 1, 3, and 5, respectively, after viral challenge (**Supplementary File 6**). Volcano plots depicting differentially expressed proteins in SARS-CoV-2-challenged MDMs compared to controls are shown in **Supplementary Figure 4**. To obtain a detailed understanding of changes in the MDM proteomic profile after the viral challenge, we performed functional and pathway enrichment analyses of differentially regulated proteins in virus-challenged MDMs at different times. Amongst total proteins altered on day 1, only 5% were involved in negative regulation of inflammatory response to an antigenic stimulus. In contrast, on days 3 and 5, 38.1% and 23.53% of altered proteins, respectively, belonged to myeloid cell activation involved in immune responses (**Figure 4A**). Immune processes of each function indicated in pie charts include, but are not limited to, activation of the innate immune response, myeloid cell activation, innate immune response activating signal transduction, and innate immune response activating cell surface receptor signaling pathway (**Figure 4A** and **Supplementary File 7**). All indicated activated immune and inflammatory responses in MDMs upon viral challenge. Additionally, viral exposure induced changes in biological processes related to the endoplasmic reticulum (ER) and mRNA on days 1 and 3 after challenge. With positive fold changes, the enriched biological processes included protein localization to ER, protein targeting to ER, regulation of mRNA metabolic process, nuclear-transcribed mRNA catabolic process, and mRNA splicing *via* spliceosome (**Supplementary File 8**). Similarly, on days 1 and 3 after the viral challenge, Reactome analysis depicted enrichment of processes related to RNA processing, with positive fold change, including rRNA processing in the nucleus and cytosol and mRNA splicing (**Supplementary File 8**). Moreover, KEGG pathway analysis showed an enrichment of ribosome function as a pathway of genetic information processing and translation on day 1. Similarly, the molecular function of a structural constituent of the ribosome was enriched with positive fold change on day 1 (**Supplementary File 8**). Interestingly, GO-term analysis showed robust enrichment of different immune responses such as complement activation, humoral immune response, adaptive immune response, and regulation of inflammatory response with negative fold changes suggesting dysregulated immune responses to SARS-CoV-2 in MDMs (**Figure 4B**). Importantly, STRING analysis of PPIs showed enrichment of IFN-related interaction networks such as “IFN signaling and positive regulation of RIG-I signaling pathway” and “IFN-α/β signaling and IFN-γ signaling”, with negative fold changes, suggesting dysregulated IFN responses induced by SARS-CoV-2 (**Figure 4B** and **Supplementary File 8**). Notably, the proteome profile of virus exposed macrophages compared to that of mock-challenged cells using SARS-CoV-2 proteome on day 5 following viral exposure (**Supplementary File 9**) showed that SARS-CoV-2 spike glycoprotein was significantly expressed (1.48-fold, p = 0.032). This confirmed the surprising virion persistence in MDMs and delayed viral clearance following the infection (**Figure 1C**.iii****). Overall, these results support transcriptomic, IFN activity, and protein evaluations that demonstrated attenuated IFN responses and delayed viral clearance in SARS-CoV-2-challenged MDMs.

### Integrated Transcriptomic and Proteomic Data Analyses of SARS-CoV-2-Challenged MDMs

The overlapping genes between the transcriptomic and proteomic datasets were identified and illustrated in **Supplementary File 10**. Venn diagrams show the number of genes identified in transcriptomic and proteomic datasets and the number of overlapped genes in both datasets at different time points following the virus challenge was calculated (**Supplementary Figure 5**). Additionally, Pearson’s product moment correlation coefficient between transcriptomic and proteomic expression fold change for 10 IFN pathway-related genes (**Supplementary File 11**) on days 1, 3, 5 following the virus challenge. These 10 genes were identified based on the transcriptomic data gene-enrichment analysis that showed that these genes are matched to their corresponding proteins in the proteomic dataset for IFN receptor activity, IFN-α/β signaling, IFN-γ signaling, regulation of IFN-α signaling, regulation of IFN-γ receptor activity, and regulation of IFN-γ receptor binding. Three genes (*HLA-A*, *STAT1*, and *TYK2*) showed negative correlation between transcription and translation data while the other genes showed positive correlation (**Table 1** and **Supplementary Figure 6**). Many are not significant, probably due to the few data points. Only IRF3 showed significant linear relationship between transcriptomic and proteomic detection. Interestingly, in accordance with the transcriptomic data, on day 5 following the viral challenge the proteomic data showed upregulated IFNAR1 and MX1 protein levels (1.67 and 1.34-fold with p = 0.0003 and 0.11, respectively). This suggests the possibility of commencement of IFN antiviral response late after viral challenge, which could restrict the viral replication in virus challenged MDMs.

**Table 1:** Pearson’s product moment correlation coefficient of IFN pathway-related genes.

Gene/Protein	Transcriptomic - Proteomic Correlation	
	Pearson product moment analysis	
	r	p value
B2M	0.8691	0.329
DDX58	0.8747	0.322
HLA-A	-0.0844	0.946
HLA-E	0.0214	0.863
ICAM1	0.6811	0.523
IFNAR1	0.5154	0.655
IRF3	0.9982	0.038
MX1	0.6539	0.546
STAT1	-0.0667	0.958
TYK2	-0.5610	0.621

r: Pearson correlation coefficient.

## Discussion

MPs are the first line of host defense against viral infection (9). These sentinel cells interact with SARS-CoV-2 to define COVID-19 pathogenesis. That includes, but is not limited to, CSS, ARDS, and multiorgan dysfunction (5). As a first line defense mechanism, the initial virus-MP interaction following the entry of the pathogenic virus into host cells represents a critical determinant for infectivity and pathogenesis (42–44). To this end, we have better defined such virus-host cell interactions and determined relationships between ACE2 receptor expression and monocyte-macrophage differentiation during restrictive SARS-CoV-2 infection without evidence of viral replication. Like other viral infections, we reasoned that monocytes would respond with an elevated expression of pro-inflammatory molecules and antiviral responses. Indeed, this has already been reported for influenza, Chikungunya, herpes, Zika, and lentiviral infections that include human immunodeficiency virus type one (HIV-1) and herpes viruses (36, 37, 45–47). Each elicits profound inflammation during early viral infection. However, unlike other known viral infections, SARS-CoV-2 infection is restrictive without evidence of viral replication and balances pro- and anti-inflammatory processes with a unique cell signature. The resulting signature defines the pathways towards the major cause for morbidity and mortality in COVID-19: the CSS leading to ARDS and other multi-organ failures. The specific lack of induction of IFN also serves to define a specific monocyte-macrophage-virus phenotype during both early and progressive infection (17, 48). The data set paralleled the lack of viral clearance noted to 14 days after SARS-CoV-2 exposures. All underline the persistence of pro-inflammatory activities in virus-challenged macrophages and the pathways for how persistence of innate immune responses could occur and lead to devastating lung damage preceding to the death of an infected host.

Herein, we show increased expression of viral receptor ACE2 during monocyte-macrophage differentiation. Interestingly, further induction of ACE2 expression by captopril, a well-known ACE inhibitor, was observed. Captopril induces a reduction of angiotensin II, which increases ACE2 expression and activity. This occurs through angiotensin type 1 receptor-dependent ACE2 internalization followed by lysosomal degradation (49). However, the observed upregulation of ACE2 expression failed to affect infection with SARS-CoV-2. Despite infection of MDMs with SARS-CoV-2 when ACE2 receptor expression peaked, the infection remained restrictive without evidence of viral replication in agreement with previous studies which showed that infection of human MDMs with SARS-CoV-2 was not productive (17, 18, 50). In the current study, the upregulation of *STAT1, IFNAR1*, and *MX1* at the transcriptome level in addition to the upregulation of both IFNAR1 and MX1 at the proteome level on day 5 after viral challenge suggests the possibility of commencement of type I IFN antiviral response at a late time point after viral exposure and the contribution of MX1 to the absence of viral replication seen with restrictive infection in SARS-CoV-2-challenged human macrophages (38). Furthermore, the previous study suggests that type-I IFNs are commonly regulated during SARS-CoV-2 infection of cell lines observed within 24 hours of virus infection (51). These data further support the results observed following the viral infection in human macrophages. The type-I IFN response was seen within one day of viral macrophage exposure. The inability of captopril to increase the susceptibility of MDM infection with SARS-CoV-2 is in accordance with a recent study indicating that treatment of human alveolar type-II pneumocytes with captopril induced upregulation of ACE2 expression and counteracted drug-induced reduction of SARS-CoV-2 spike protein entry (34). This occurred through inhibition of A disintegrin and metalloprotease 17 (ADAM17), which has been shown to play an essential role in ACE2 shedding and viral entry into the cells (52). Nonetheless, viral particles were present inside macrophages up to two weeks after the viral challenge underscoring any continuity of immune responses.

SARS-CoV-2 restrictive infection without evidence of viral replication likely results from intracellular mechanisms induced upon MP activation. Human coronaviruses can infect human peripheral blood mononuclear cells leading to cell activation and aberrant production of pro-inflammatory mediators with increased chemoattraction (53–55). In particular, virus-exposed monocytes-macrophages can serve as perpetrators for virus-induced inflammatory responses within different body organs, as seen by the abundance of pro-inflammatory macrophages in bronchoalveolar lavage fluid obtained from severe COVID-19 cases (56). The MP pro-inflammatory factors can contribute to local tissue inflammation and systemic inflammatory responses that characterize cytokine storm (9). In the current study, the transcriptomic profile of SARS-CoV-2-challenged MDMs demonstrated increased mRNA expression of multiple pro-inflammatory molecules, including *IL-6, TNF-α, IL-1α, IL17A, IL8, CSF2, CCL2, CCL5, NLRP3*, and *IL-1β* upon the virus exposure. Our data confirmed previous reports which showed excessive production of pro-inflammatory cytokines and chemokines in the SARS-CoV-2-exposed MPs (17, 18, 50). In humans, TLR8 is more prominently present in myeloid lineage cells such as monocytes and neutrophils (57). The prominent ligand for TLR7 and TLR8 is viral single-stranded RNA (ssRNA) (58) and, more specifically for TLR8, its RNAse T2 degradation products (59). GU-rich ssRNA derived from SARS-CoV-2, SARS-CoV-1, and HIV-1 was found to trigger a TLR8-dependent pro-inflammatory cytokine response from human macrophages in the absence of pyroptosis, with GU-rich RNA from the SARS-CoV-2 spike protein triggering the greatest inflammatory response (60). In our study, the slight upregulation of *TLR8* gene expression on day 5 following viral challenge agrees with a recent study demonstrating that lung TLR8 contributes to the cytokine storm during COVID-19 disease (61). In parallel, our data displayed enrichment of different inflammation-related pathways such as IL-17, NF-κB, and TNF signaling pathways, following viral exposure. IL-17 is known for its pivotal role in inducing and mediating pro-inflammatory responses and its involvement in different inflammatory autoimmune diseases (62). NF-κB serves as a central mediator of inflammation since the DNA binding site for NF-κB was found in the promoter regions of multiple pro-inflammatory molecules (63). Thus, the activation of NF-κB with other pro-inflammatory transcription factors leads to the transcription of several pro-inflammatory molecules such as IL-1β, inducible nitric oxide synthase (iNOS), and TNF-α (64, 65). In addition, the transcriptome of SARS-CoV-2-challenged MDMs showed enrichment of multiple immune-related biological processes, molecular functions, and signaling pathways such as positive regulation of immune response, cell surface receptor signaling pathway, cytokine activity, chemokine activity, TLR signaling pathway, and NOD-like receptor signaling pathway, demonstrating cell activation of MDMs after viral exposure. Notably, the proteome of virus-challenged macrophages revealed the dysregulation of different immune processes such as regulation of inflammatory response, complement activation, and linked adaptive and humoral immune responses. Overall, our findings demonstrate that MDM activation by SARS-CoV-2 is associated with exaggerated inflammatory responses and dysregulated immune activities.

Significantly, SARS-CoV-2 induced an attenuated MP IFN response, as the transcript expression of type I (IFN-α1 and IFN-β1) and type II (IFN-γ) IFNs in the virus-challenged cells increased early after exposure but returned to or below normal levels at later times. Our findings are consistent with previous reports depicting a dysregulated IFN response in SARS-CoV-2-challenged MDMs and alveolar macrophages (17, 48). This attenuated IFN response provides a possible mechanism for delayed viral clearance from cells up to 2 weeks after infection. Similarly, our study showed the inability of SARS-CoV-2 to induce IFN activity or production in MDMs, as illustrated by the failure of culture supernatants of infected cells to provide protection against the cytopathic effects induced by VSV in MDBK cells as well as unchanged levels of IFN-α and IFN-β after the virus challenge. These findings follow a recent study that demonstrated the absence of IFN induction in the alveolar macrophages challenged with SARS-CoV-2 (48). A possible explanation is the presence of a cap structure on the viral genome. The *Coronoviridae* family contains this structure enabling the virus to evade recognition by PRRs and prevent the host innate immune response mediated by the RIG-I/mitochondrial antiviral-signaling (MAVS) pathway that recognizes ssRNAs without a cap structure (66, 67). Moreover, different SARS-CoV-2 proteins were found to antagonize type I IFN production through other mechanisms (68). SARS-CoV-2 nonstructural protein 6 (nsp6) binds TANK binding kinase 1 (TBK1) to suppress IRF3 phosphorylation, and nsp13 binds and blocks TBK1 phosphorylation. In addition, open reading frame 6 (ORF6) binds importin Karyopherin α 2 to inhibit IRF3 nuclear translocation, and ORF7b prevents STAT1 phosphorylation and nuclear translocation, consequently inhibiting the transcription of multiple IFN stimulated genes (ISGs) which possess antiviral functions (68). In parallel, the negative fold change of IFN-related interaction networks: “IFN signaling and positive regulation of RIG-I signaling pathway” and “IFN-α/β signaling and IFN-γ signaling” indicates dysregulated IFN responses in the SARS-CoV-2-challenged MDMs. Thus far, our data suggest an aberrant IFN response induced by SARS-CoV-2 in human MDMs. Early IFN induction was halted at the transcriptional level one day post-infection. It did not proceed to the translational level because of SARS-CoV-2 proteins found to antagonize type I IFN production (68). Thus, we postulate that aberrant IFN responses in the face of a robust inflammatory environment presage a lack of viral infection control and multi-organ damage in COVID-19 (3).

In summary, we demonstrate that persistence of SARS-CoV-2 components in macrophage infection triggers a unique signature of inflammatory responses despite the absence of viral replication. This includes combinations of restrictive infection with delayed viral clearance, excessive pro-inflammatory cytokine and chemokine production, and attenuated IFN responses. Each and all imply dysfunction of the innate immune system after viral exposure. Transcriptomic and proteomic profiles of SARS-CoV-2-challenged MPs provide signatures for understanding COVID-19 pathogenesis. The lack of adequate antiviral innate immune responses in the midst of cytokine storm heralds an absence of viral infection control that exacerbates clinical manifestations and contributes to end-organ damage for COVID-19-related morbidities and mortalities.

## Data Availability Statement

The raw transcriptomic data for transcriptomic assays are openly available in figshare at https://doi.org/10.6084/m9.figshare.16550286.v1 and doi.org/10.6084/m9.figshare.16451862.v3.

## Author Contributions

MMA: Investigation, Methodology, Validation, Visualization, Formal analysis, Data curation, Writing-original draft, review and editing; PY: Investigation, Methodology, Formal analysis, Data curation, Writing-review and editing; JM: Investigation, Methodology, Data curation, Writing-review and editing; KEO: Validation, Visualization, Writing-review and editing; FS: Investigation, Validation, Writing-review and editing; VK: Investigation, Formal analysis, Writing-review and editing; YZ: Investigation, Validation, Data curation, Writing-review and editing; JL: Investigation, Formal analysis, Writing-review and editing; KP: Investigation, Validation, Writing-review and editing; AA: Investigation, Validation, Visualization, Writing-review and editing; SNB: Supervision, Visualization, Writing-review and editing; RLM: Conceptualization, Methodology, Supervision, Writing-review and editing; HEG: Conceptualization, Funding Acquisition, Methodology, Resources, Supervision, Writing-original draft, review and editing; All authors have read and approved the final version of the manuscript. All authors contributed to the article and approved the submitted version.

## Funding

The work was supported, in part, by the University of Nebraska Foundation, which includes donations from the Carol Swarts, M.D. Emerging Neuroscience Research Laboratory, the Margaret R. Larson Professorship, the Frances and Louie Blumkin, and the Harriet Singer Research Donations. We thank Dr. Bradley Britigan, Dean of the College of Medicine at UNMC, for providing funds to support the SARS-CoV-2 studies. The research also received support from National Institutes of Health grants PO1 DA028555, R01 NS36126, PO1 MH64570, P30 MH062261, P20 GM113126, R01 AG043540, and 2R01 NS034239. We also thank the INBRE grant support from 2P20GM103427 for infrastructure research support.

## Conflict of Interest

Author HEG is a co-founder of Exavir Therapeutics, Inc. who is developing antiviral and elimination therapies for HIV/AIDS and other viral infections. 

The remaining authors declare that the research was conducted in the absence of any commercial or financial relationships that could be construed as a potential conflict of interest.

## Publisher’s Note

All claims expressed in this article are solely those of the authors and do not necessarily represent those of their affiliated organizations, or those of the publisher, the editors and the reviewers. Any product that may be evaluated in this article, or claim that may be made by its manufacturer, is not guaranteed or endorsed by the publisher.

## Supplementary Material

Supplementary Figure 1 Representative histograms of ACE2, CD14, and CD16 in human monocyte-macrophages. Expression of SARS-CoV-2 cell entry receptor ACE2 **(A)** and phenotypic surface markers CD14 **(B)** and CD16 **(C)**, during monocyte-macrophage differentiation was analyzed by flow cytometry in absence or presence of captopril. All experiments were done at least twice with representative images (n=3 donors). w/o: without.

Supplementary Figure 2 Kinetic growth of SARS-CoV-2 in human monocyte-macrophage. MOIs used for SARS-CoV-2 challenge in MDMs were 0.001, 0.01, and 0.1. Number of virus genome equivalents/ml was measured in culture supernatants by RT-qPCR on day 11 (n=4 donors).

Supplementary Figure 3 Fold changes of immune response genes in SARS-CoV-2-challenged MDMs. Heat map of fold changes in the expression of 84 genes specific for human innate and adaptive immune responses in SARS-CoV-2-challenged MDMs compared to mock-challenged MDMs at different time points after the infection, determined using RT2 Profiler Human Innate and Adaptive Immune Response 96-well Array. Fold changes were determined *via* Qiagen’s RT2 Profiler analysis software (n=4 donors).

Supplementary Figure 4 Differential proteomic analysis of SARS-CoV-2-challenged MDMs. Volcano plots showing the fold change plotted against the P value highlighting significantly changed proteins (red – upregulation and green – downregulation; p ≤ 0.05 and an absolute fold change ≥ 2) in SARS-CoV-2-challenged MDMs compared to mock-challenged MDMs at different time points (n=4 donors). The vertical lines correspond to the absolute fold change of 2, and the horizontal line represents a p value of 0.05.

Supplementary Figure 5 Venn diagrams showing overlap in quantified genes and proteins of SARS-CoV-2-challenged MDMs. Overlapping genes between transcriptomic and proteomic data (n=4 donors) were identified at defined time points after viral challenge.

Supplementary Figure 6 Correlation analyses of IFN pathway-related genes. For all correlation analyses, 95% confidence intervals were used. Data are displayed as scatter plots and correlations were determined using Pearson product moment correlation coefficients and were adjusted for FDR.
