# Supplementary figures and images for: Defining the Innate Immune Responses for SARS-CoV-2-Human Macrophage Interactions
Source: Front Immunol. 2021 Oct 4;12:741502. doi: 10.3389/fimmu.2021.741502 (PMC8521106; doi:10.3389/fimmu.2021.741502)

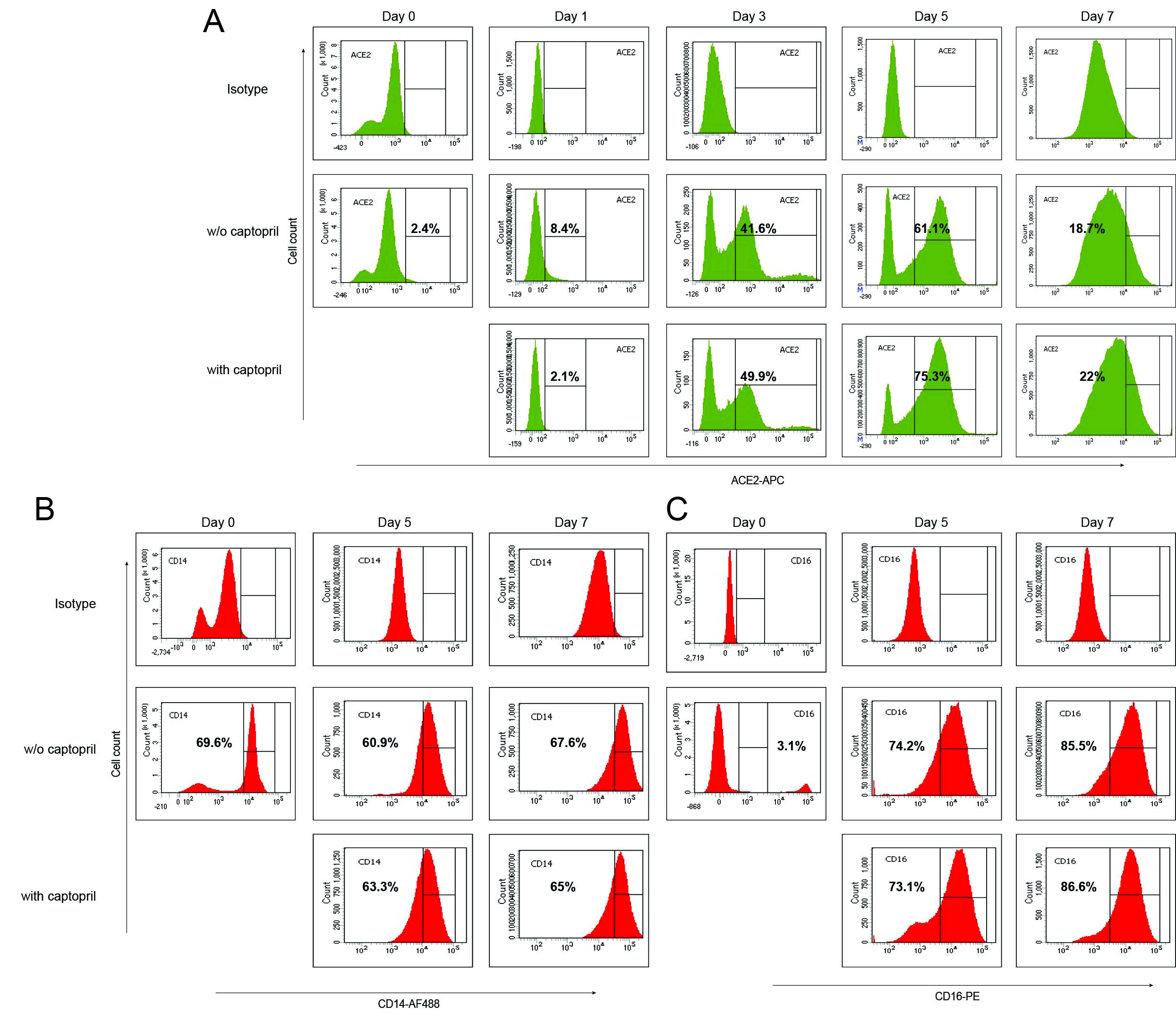

Supplement: Supplementary Figure 1 — Representative histograms of ACE2, CD14, and CD16 in human monocyte-macrophages. Expression of SARS-CoV-2 cell entry receptor ACE2 (A) and phenotypic surface markers CD14 (B) and CD16 (C), during monocyte-macrophage differentiation was analyzed by flow cytometry in absence or presence of captopril. All experiments were done at least twice with representative images (n=3 donors). w/o: without. [file Image_1.jpeg]

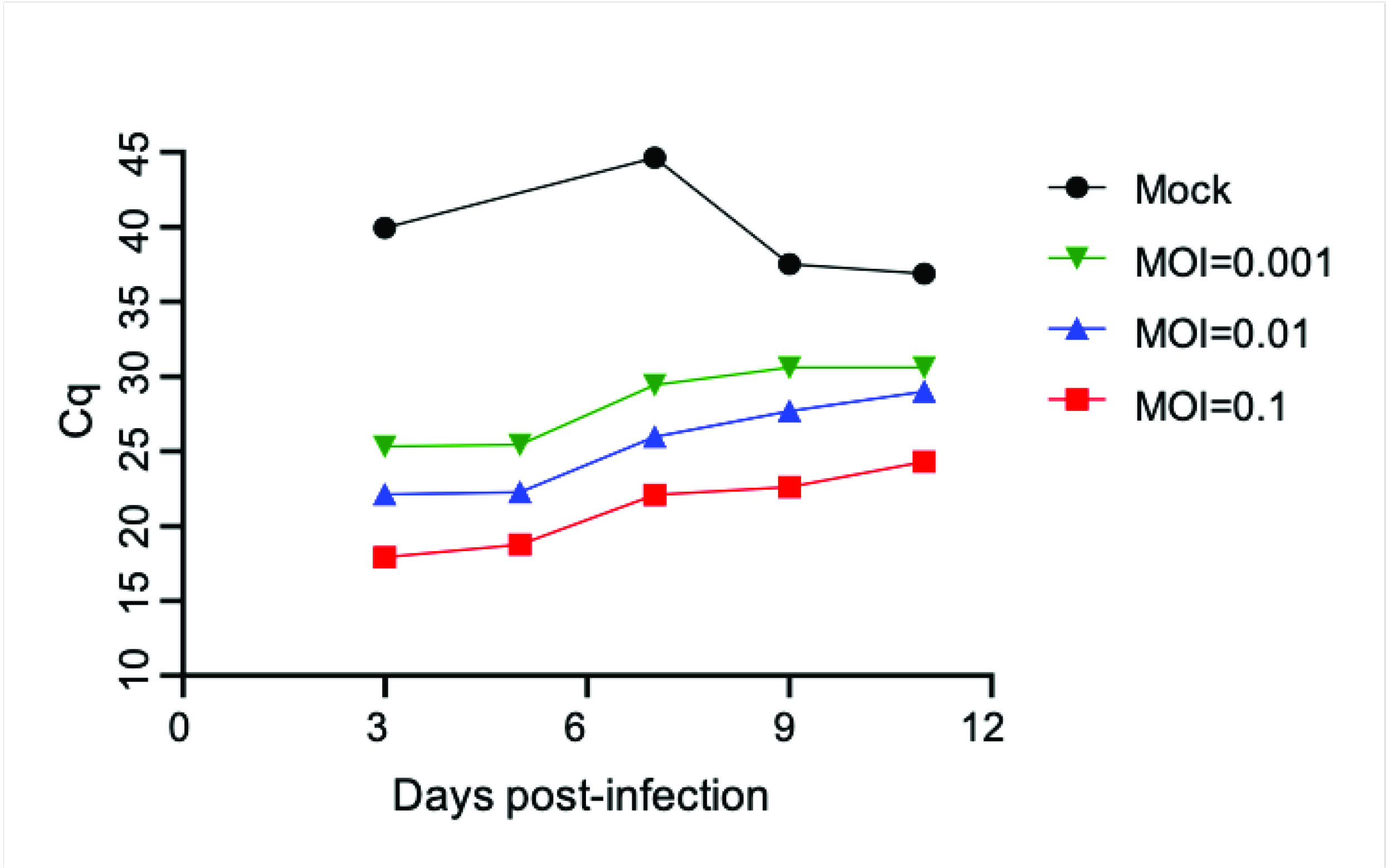

Supplement: Supplementary Figure 2 — Kinetic growth of SARS-CoV-2 in human monocyte-macrophage. MOIs used for SARS-CoV-2 challenge in MDMs were 0.001, 0.01, and 0.1. Number of virus genome equivalents/ml was measured in culture supernatants by RT-qPCR on day 11 (n=4 donors). [file Image_2.jpeg]

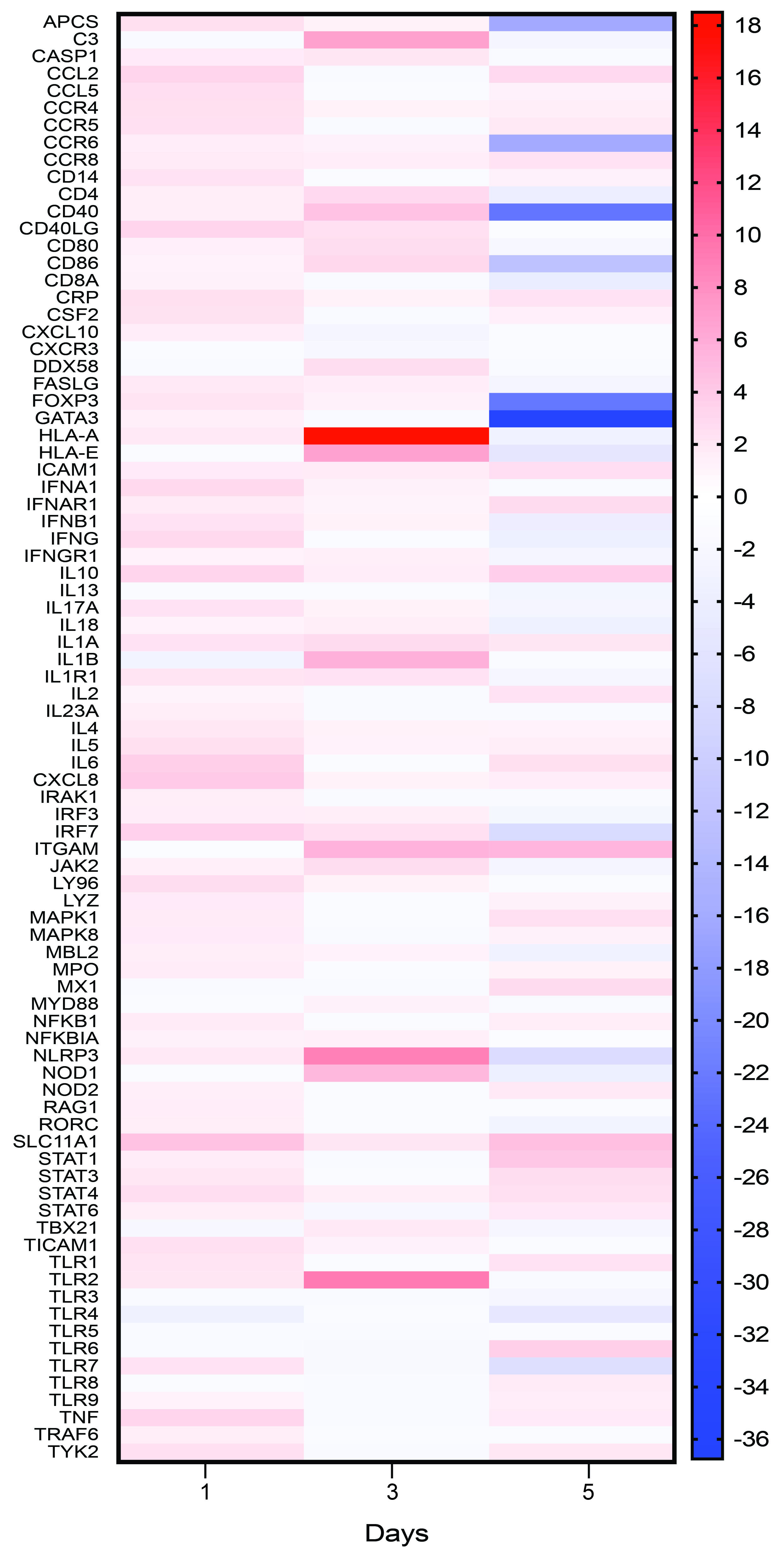

Supplement: Supplementary Figure 3 — Fold changes of immune response genes in SARS-CoV-2-challenged MDMs. Heat map of fold changes in the expression of 84 genes specific for human innate and adaptive immune responses in SARS-CoV-2-challenged MDMs compared to mock-challenged MDMs at different time points after the infection, determined using RT2 Profiler Human Innate and Adaptive Immune Response 96-well Array. Fold changes were determined via Qiagen’s RT2 Profiler analysis software (n=4 donors). [file Image_3.jpeg]

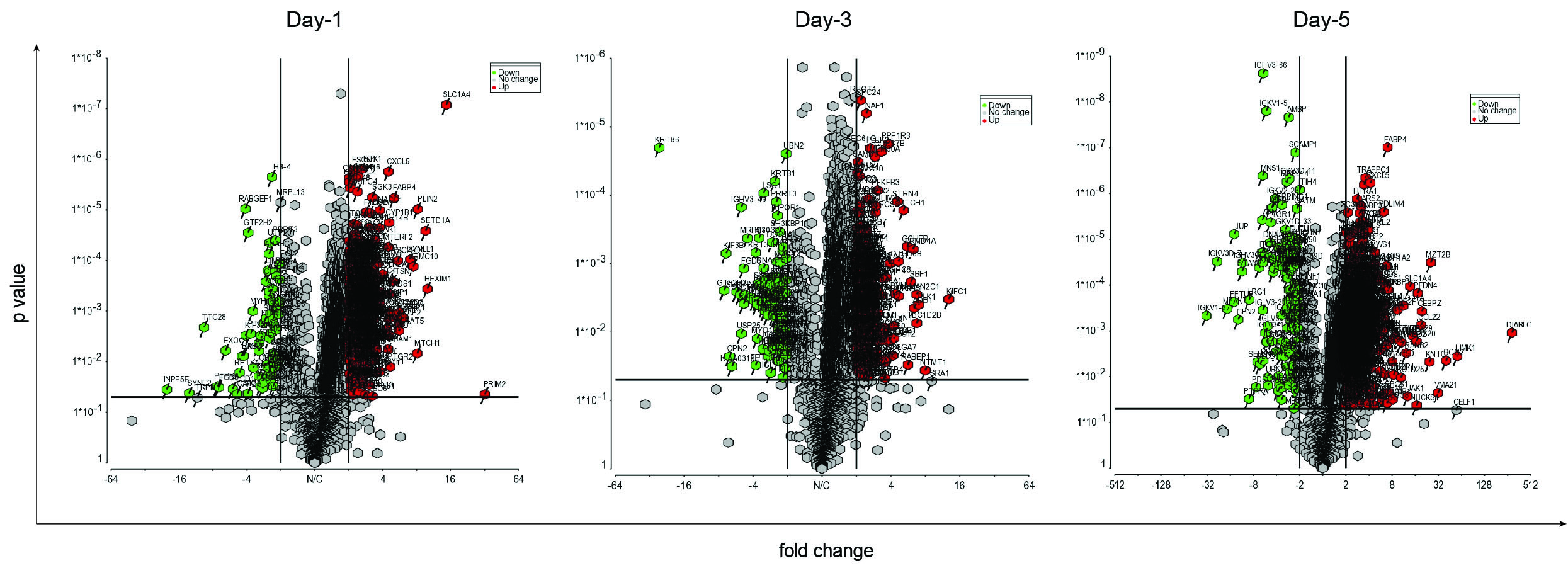

Supplement: Supplementary Figure 4 — Differential proteomic analysis of SARS-CoV-2-challenged MDMs. Volcano plots showing the fold change plotted against the P value highlighting significantly changed proteins (red – upregulation and green – downregulation; p ≤ 0.05 and an absolute fold change ≥ 2) in SARS-CoV-2-challenged MDMs compared to mock-challenged MDMs at different time points (n=4 donors). The vertical lines correspond to the absolute fold change of 2, and the horizontal line represents a p value of 0.05. [file Image_4.jpeg]

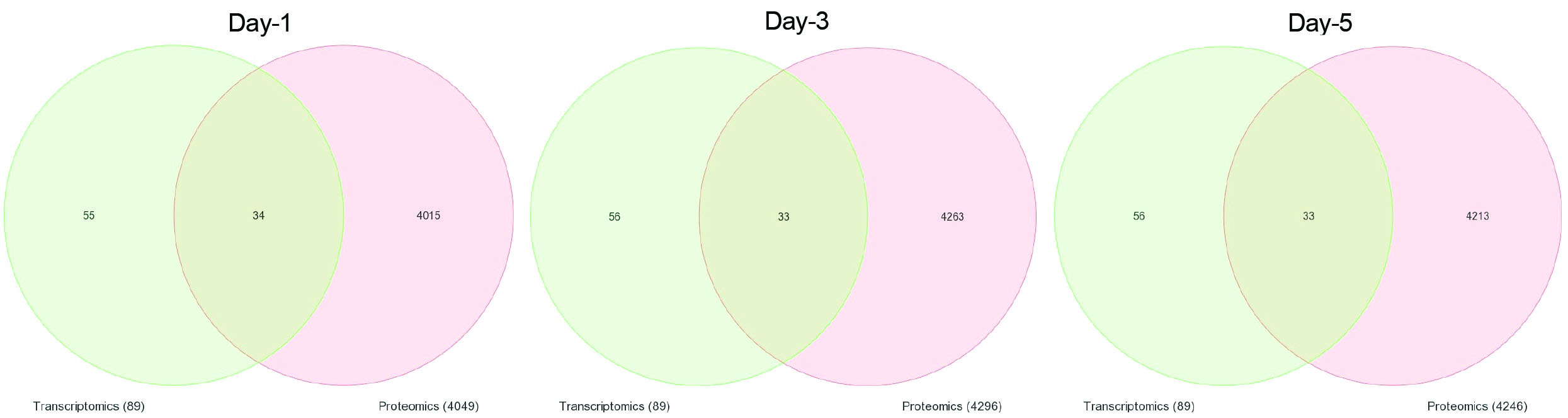

Supplement: Supplementary Figure 5 — Venn diagrams showing overlap in quantified genes and proteins of SARS-CoV-2-challenged MDMs. Overlapping genes between transcriptomic and proteomic data (n=4 donors) were identified at defined time points after viral challenge. [file Image_5.jpeg]

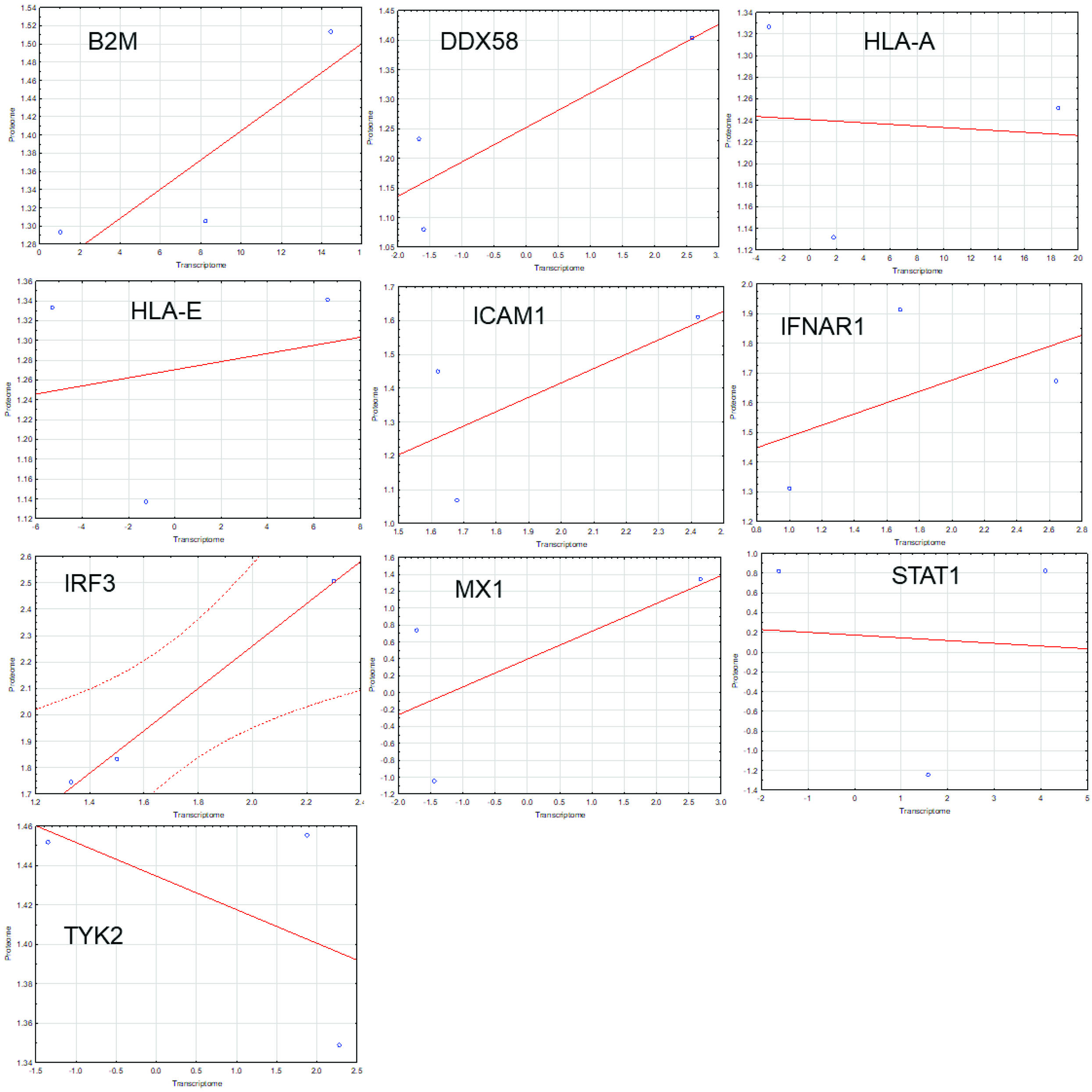

Supplement: Supplementary Figure 6 — Correlation analyses of IFN pathway-related genes. For all correlation analyses, 95% confidence intervals were used. Data are displayed as scatter plots and correlations were determined using Pearson product moment correlation coefficients and were adjusted for FDR. [file Image_6.jpeg]
